# Symptoms and Association with Health Outcomes in Relapsing-Remitting Multiple Sclerosis: Results of a US Patient Survey

**DOI:** 10.1155/2014/203183

**Published:** 2014-09-23

**Authors:** Angela E. Williams, Jeffrey T. Vietri, Gina Isherwood, Armando Flor

**Affiliations:** ^1^MedImmune, Global Payer Evidence, Riverside 2 Building, Granta Park, Cambridge, Cambridgeshire CB32 6GH, UK; ^2^Kantar Health, Health Outcomes Practice, Paleocapa 7, 20121 Milan, Italy; ^3^Kantar Health, Health Outcomes Practice, The Kirkgate, 19-31 Church Street, Epsom, Surrey KT17 4PF, UK; ^4^MedImmune, Clinical Development, 1 MedImmune Way, Gaithersburg, MD 20878, USA

## Abstract

*Background*. A variety of symptoms have been reported, but the prevalence of specific symptoms in relapsing-remitting multiple sclerosis (RRMS), how they are related to one another, and their impact on patient reported outcomes is not well understood. *Objective*. To describe how symptoms of RRMS cooccur and their impact on patient-reported outcomes. *Methods*. Individuals who reported a physician diagnosis of RRMS in a large general health survey in the United States indicated the symptoms they experience because of RRMS and completed validated scales, including the work productivity and activity impairment questionnaire and either the SF-12v2 or SF-36v2. Symptom clusters were identified through hierarchical cluster analysis, and the relationship between clusters and outcomes was assessed through regression. *Results*. Fatigue, difficulty walking, and numbness were the most commonly reported symptoms. Seven symptom clusters were identified, and several were significantly related to patient reported outcomes. Pain, muscle spasms, and stiffness formed a cluster strongly related to physical quality of life; depression was strongly related to mental quality of life and cognitive difficulty was associated with work impairment. *Conclusions*. Symptoms in RRMS show a strong relationship with quality of life and should be taken into consideration in treatment decisions and evaluation of treatment success.

## 1. Introduction

Relapsing-remitting multiple sclerosis (RRMS) is associated with a variety of symptoms, but neither the prevalence of specific symptoms nor how they are related to one another is well understood. While slowing disease progression is usually the first priority of treatment, patient well-being and quality of life (QoL) may be profoundly affected by experience of symptoms. Therefore, understanding how symptoms cooccur and how the wellbeing of the patient is affected by symptoms is important for understanding patients with this condition.

In RRMS and other diseases with numerous and disparate symptoms, it may be difficult to consider symptoms individually. If multiple symptoms are related to one another, it may be useful to consider those symptoms as a symptom cluster, defined as “3 or more concurrent symptoms that are related to each other” [1, (page 465)]. Indeed, research has documented that fatigue, depression, and anxiety are observed together at higher-than-chance levels among individuals with multiple sclerosis (MS) and vary together [2, 3]. Several studies have documented the usefulness of considering pain, fatigue, and depression as a cluster to explain level of physical activity or QoL of MS patients [4–6] or explored the relationship between pain severity and other MS symptoms [7]. Experience of specific symptoms, such as pain, depression, and fatigue, has been associated with greater impairment at work [8] as well as health-related QoL (HRQoL) [9, 10]. In particular, pain, fatigue, and depression considered as a cluster have been demonstrated to impact HRQoL [11, 12] as well as physical activity [5, 13].

Documenting the relationship between a group of symptoms and outcomes in RRMS is useful, but an understanding of the relationship between symptoms themselves is still lacking. Existing studies of symptom clusters in MS and RRMS rarely investigate whether the cluster used is the one with the most explanatory power or whether symptoms outside of the few most common ones might form a second cluster; truly exploratory studies are rare. The few published studies that come closest to describing the clustering of the symptoms themselves have assessed the cooccurrence of relatively few symptoms [2, 3, 14–17], qualitative research with limited numbers of patients [18], or explored the impact of incorporating additional symptoms in a single cluster on explaining outcomes [5, 7]. To our knowledge, there is no quantitative, data-driven identification of symptom clusters described in the literature. Likewise, because much of the previous work recruited patients through MS clinics, these studies may not describe the symptoms of individuals with MS who are treated elsewhere or, or not at all.

The purpose of the current study was threefold: first, to describe the symptoms or RRMS from the patients' perspective using a large, representative sample recruited through the general population rather than MS-specific means; second, to explore how symptoms cluster together in patients; and third, to assess the relationship between symptom clusters and health outcomes, including HRQoL and work impairment.

## 2. Materials and Method

Data came from the US National Health and Wellness Survey (NHWS; Kantar Health, New York, NY, USA). This is an annual, cross-sectional study of more than 70,000 adults aged 18 years or older. The sample was selected using a stratified random sample framework (with quotas based on gender, age, and white/nonwhite race) from a consumer panel of more than one million US residents. The panel recruits its members through opt-in emails, coregistration with other survey panels, e-newsletter campaigns, and online banner placements. All subjects provided informed consent and the NHWS was approved by Essex Institutional Review Board (Lebanon, NJ, USA). Data from the 2011 (*n* = 75,000) and 2012 (*n* = 71,157) surveys were combined to increase the number of patients available for analysis. No respondents with RRMS participated in both the 2011 and 2012 surveys. The survey was administered via the Internet, and all measures were by self-report.

### 2.1. Measures

#### 2.1.1. Demographics and General Health

Age, sex, race, education, possession of health insurance, household income, cigarette smoking, frequency of alcohol use, and self-reported diagnosis of a variety of medical conditions were assessed in NHWS. Diagnoses were used to calculate the Charlson comorbidity index (CCI) [19] for use as a covariate in analyses of health outcomes.

#### 2.1.2. MS Characteristics

Respondents characterized their MS by type, symptoms, and severity. Respondents also indicated whether they were currently using a prescription for their MS and if not, whether they had ever received prescription treatment. Only those who indicated RRMS were included in the analyses. Experience of symptoms was assessed using a checklist of 24 possible symptoms, and respondents indicated which they experience because of their multiple sclerosis. Severity of multiple sclerosis was self-assessed as mild, moderate, or severe.

#### 2.1.3. Health-Related Quality of Life

Health status was collected using the revised Medical Outcomes Study Short Form Health Surveys. These generic health surveys are among the most commonly used patient-reported outcomes measures. The 2012 survey included the 36-item version (SF-36v2) [20], and the 2011 survey included the 12-item version (SF-12v2) [21]. The SF-12v2 is a shortened version of the SF-36v2 and reports on the same health concepts with the same norms, so scores from the two versions were combined for analysis. The current project focuses on three summary values. The first is a single-item overall rating of health on a 5-point scale from poor to excellent used in population health studies. The other two are the physical component summary (PCS) and mental component summary (MCS) scores, respectively. MCS and PCS scores have an average of 50 and standard deviation of 10 in the US population, and higher scores indicate better functional health. Responses to the SF surveys were also used to calculate health state utilities using the SF-6D algorithm, a preference-based index of HRQoL general population values [22]. SF-6D scores are interpreted on a theoretical 0-1 scale, with an empirical floor of 0.3 (i.e., scores below 0.3 are not possible). Higher scores indicate better quality of life.

#### 2.1.4. Impairment due to Health

The Work Productivity and Activity Impairment-General Health (WPAI-GH) questionnaire was used to measure the impact of health on activities [23]. Four metrics of impairment over the previous seven days were calculated: absenteeism (the percentage of work time missed because of one's health), presenteeism (the percentage of impairment experienced while at work because of one's health), overall work impairment (a combination of absenteeism and presenteeism), and activity impairment (impairment in daily nonwork activities because of one's health). Only respondents who reported full-time, part-time, or self-employment provided data for work-related metrics, and all respondents provided data for activity impairment.

### 2.2. Analysis

To assess the relationship between the presence of symptoms and perceived severity of MS, the frequency of each symptom was compared across levels of severity. Moderate and severe patients were considered as a single group, due to relatively few reporting severe MS, and compared against mild patients using chi-square tests.

Clusters of symptoms were identified through hierarchical cluster analysis, using least squared distance, which provides a map of how to organize symptoms from the highest level, where all symptoms are considered as a single group, to the lowest, where each is considered alone. To ensure that the results were replicable, the results of the analysis were compared to the results of a second analysis based on a randomly selected subsample incorporating half of the respondents.

Relationships between symptom clusters and outcomes were modelled using one regression per outcome. Linear regressions were used for HRQoL, as the distributions were sufficiently normal. Binary logistic regression was used for absenteeism, which was modelled as any versus none because of convergence problems when the amount of absenteeism was modelled, as was self-reported MS severity (mild versus moderate or severe). Generalized linear models using a negative binomial distribution and a log-link were used for presenteeism and overall work impairment because of a pronounced positive skew in the distributions of those variables. Symptom clusters were included in the models as dichotomous variables, wherein respondents endorsing less than the median number of symptoms in a cluster were coded as 0 and those with at least the median number of symptoms were coded as 1 for that cluster (coding of variables is available from the corresponding author). Covariates included age, length of MS diagnosis, sex, race (white versus nonwhite), household income, whether the individual currently smokes cigarettes and drinks alcohol, and CCI. An alpha error rate of 5% (two-sided) was adopted for all analyses.

## 3. Results

### 3.1. Patient Characteristics

A total of 447 respondents reported RRMS. Demographic and disease characteristics are presented in Table 1. The sample was primarily female, white, and educated, with a mean age of 49.3 years. Almost half characterized their MS as mild, and most rated their MS as moderate, with a few who considered their MS as severe. Over two-thirds were overweight/obese (67.1%) and 23% were current smokers. Average length of diagnosis was 12.3 years and the majority of patients were currently being treated, mostly with disease modifying medications.

### 3.2. Symptoms

The proportion of respondents reporting each symptom is presented in Table 2. The most commonly reported symptoms were fatigue, difficulty balancing or walking, numbness, pain, and difficulty remembering, all experienced by more than half of the respondents. Those who considered their MS mild reported fewer symptoms on average (6.0 versus 9.7 for moderate/severe patients, *P* < 0.001), but every symptom included in the survey was endorsed by at least two patients reporting mild MS. More than 50% of such “mild” patients each reported fatigue, difficulty balancing or walking, or numbness. However, most symptoms were significantly more likely among those who considered their disease to be moderate or severe. The only symptoms not more likely among moderate and severe than mild respondents were fatigue, mood swings, vision problems, diarrhea, and seizures.

### 3.3. Clustering of Symptoms

**Figure 1:**
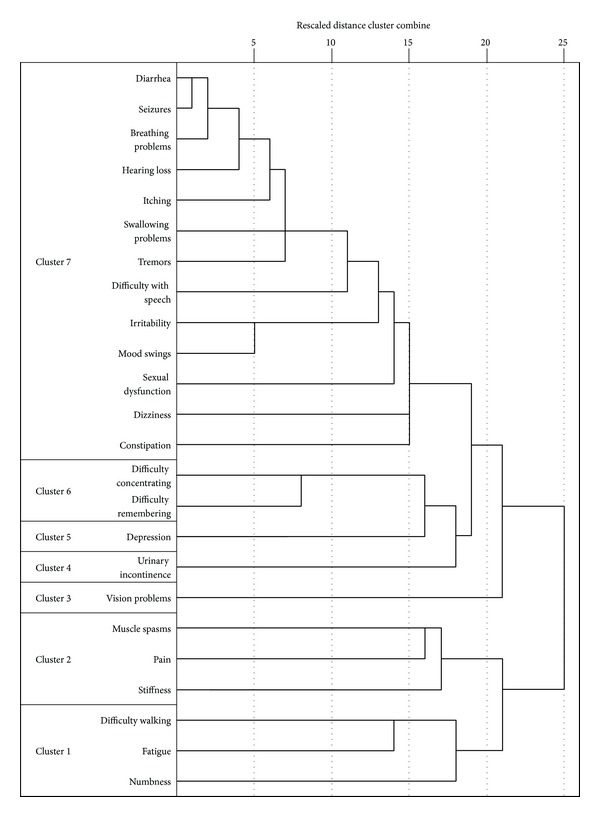
Dendrogram of symptoms in RRMS. Note: symptoms whose lines intersect towards the left of the figure are more closely related than those whose lines intersect further to the right.

A dendrogram illustrating the relationships between the symptoms according to the cluster analysis is presented in Figure 1. When only two clusters are considered, the symptoms are split into numbness, fatigue, difficulty balancing or walking, stiffness, pain, and muscle spasms in one cluster, and the remaining symptoms (breathing problems, constipation, difficulty concentrating, diarrhea, depression, difficulty remembering, difficulty with speech, dizziness, hearing loss, irritability, itching, mood swings, sexual dysfunction, swallowing problems, seizures, tremors, urinary incontinence, and vision problems) in the other. When considered as four clusters, walking, fatigue, and numbness split from stiffness, pain, and muscle spasms, while vision problems split from the remaining symptoms. A decision was made to include cognitive symptoms (difficulty concentrating and difficulty remembering) as their own cluster and likewise to treat depression independently. The arrangement of the symptoms in the dendrogram after separation of these variables also resulted in urinary incontinence being considered as a cluster of one symptom. This resulted in a set of seven symptoms/symptom clusters for inclusion in the regression models of outcomes, which preserved the general structure of the clusters described by the dendrogram (all symptoms in each cluster were contiguous).

### 3.4. Clusters and Outcomes

**Figure 2:**
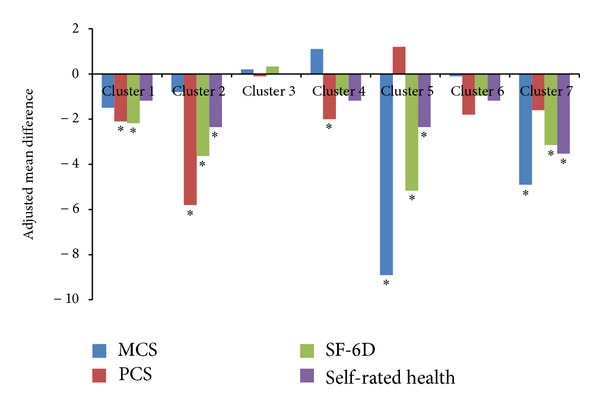
Regression-adjusted decrements in health-related quality of life associated with symptom clusters. Note: bars represent mean adjusted difference between presence of a symptom (clusters 3, 4, and 5) or more numerous symptoms in the cluster (clusters 1, 2, 4, and 7) relative to absence or less numerous symptoms in the cluster, respectively. Cluster labels are presented in Figure 1. SF-6D scores and self-rated health have been rescaled to allow for presentation with MCS and PCS scores. *P < 0.05.

The relationship between the symptom clusters and different components of HRQoL are depicted in Figure 2. After taking into account the contribution of the covariates and other symptom clusters, the cluster including walking, fatigue, and numbness (cluster 1) was related to both PCS scores (−2.1 points) and health utility scores (−.027 points). The cluster including pain, muscle spasms, and stiffness (cluster 2) had the largest impact on PCS (−5.8 points) and was also associated with poorer health utility scores (−.045 points) and global self-rated health (−.2 points). Neither vision problems (cluster 3) nor difficulty concentrating or remembering (cluster 6) was significantly associated with HRQoL measures in the regressions. Urinary incontinence or urgency (cluster 4) was associated only with lower PCS scores (−2.0 points). Depression (cluster 5) was strongly related to MCS (8.9 points), health utilities (−.064 points), and global self-rated health (−.2 points), but not PCS. The cluster including all other symptoms (cluster 7) was associated with sizable HRQoL decrements in MCS scores (−4.9 points), health utility scores (−.039 points), and global self-rated health (−.3 points).

Analyses for absenteeism, presenteeism, and overall work impairment included only the 41% of respondents who were employed. The cluster of cognitive symptoms (cluster 6; difficulty concentrating and difficulty remembering) was associated with greater adjusted odds of absenteeism (OR = 3.3, *P* < 0.05), a greater amount of presenteeism (an absolute increase of 15.8%, *P* < 0.05), and more overall work impairment (absolute increase of 19.4%, *P* < 0.05). No other clusters were significant. Impairment in daily activities was associated with cluster 2 (pain, stiffness, and spasms; 13.6%, *P* < 0.001), cluster 5 (depression; 7.4%, *P* < 0.05), and other symptoms (cluster 7; 7.4%, *P* < 0.05). Higher adjusted odds of reporting moderate or severe MS relative to mild MS were associated with the pain cluster (OR = 4.0, *P* < 0.0001) and there was a trend for cluster 7 (symptoms not included in other clusters) to have higher odds of moderate or severe MS severity (OR = 1.7, *P* = 0.051).

## 4. Discussion

The present study demonstrated that patients' perceptions of their MS and important patient-centric outcomes, such as quality of life, were strongly related to the experience of symptoms. These symptoms may be understood as clusters of symptoms, and the seven clusters of symptoms described in the present study were significantly associated with health-related quality of life, impairment in daily activities, work impairment, and perceived severity of MS.

The present study included 24 possible symptoms, many more than those typically discussed in the RRMS literature. Each symptom was endorsed by at least a few RRMS patients, illustrating the varied nature of patient experience with this condition. The present study also differed from other studies of symptoms in RRMS in the source of respondents, as these patients are typically studied because of a relationship with a specialized MS center, such as an academic hospital, MS clinic, or national MS society, limiting the pool of respondents to those who are engaged with their disease or being treated through specialized facilities. In contrast, the present sample was recruited through a large health survey designed to be representative of the US population as a whole, providing a look at what may be a more “real world” patient population.

A clear message from the current study is that the number and type of symptoms are important in determining patients' perception of disease severity. More severe patients reported a greater number of symptoms on average, and the marginal association between the large cluster of less-prevalent symptoms and self-reported severity also supports the idea that more numerous symptoms drive perceived severity.

A novel aspect of the present study is the exploratory cluster analysis of self-reported symptoms in RRMS. Previous work discussing clusters has explored the association between previously hypothesized clusters and outcomes rather than identifying clusters per se. The clusters then emerged from our analysis were slightly different than what has been the focus of the literature to this point. Previous work incorporating symptom clusters in MS focuses on pain, fatigue, and depression as a single cluster [4, 5, 11], while the present analysis placed each of these three individual symptoms in separate clusters. This is partially due to the structure of the different studies; previous studies have sought to use a single cluster of symptoms to explain outcomes, while this study attempted to identify clusters. It is important to note that there are methodological differences in measurement of the clusters between previous studies and the present analysis, as the current study assessed the presence of symptoms without an explicit timeframe, while previous research on symptom clusters has typically assessed symptoms through scales measuring current severity of individual symptoms.

This study is also different from a previous application of cluster analysis to MS patients, which clustered patients rather than their symptoms [12]. Although that study found a relationship between clusters and outcomes, the resulting groups did not provide much detail on the relationship between specific symptoms and outcomes. The clusters included here are tied to specific aspects of health related quality of life, which indicate how much a patients' health interferes with their physical, mental, and social functioning. The cluster including pain was by far the most closely related to physical functioning, while depression was the most closely associated with mental and emotional well-being. These relationships were large according to guidelines for clinical significance on SF-12v2/SF-36v2 presented in literature [24, 25]. However, it is also important to note that the 7th cluster, which served primarily as an indicator of more numerous symptoms, also had a significant and substantial relationship with mental quality of life and the largest relationship with self-rated health, despite the other symptom clusters also being included in the same regression. Interestingly, the cluster that includes difficulty walking—the primary indicator of disease status in the commonly used Expanded Disability Status Scale (EDSS; [26])—was a significant predictor of physical well-being and health utility scores but did not itself account for a clinically meaningful decrement in HRQoL.

### 4.1. Limitations

There are a number of limitations to the current study that should be considered alongside the results. As the NHWS is a self-report survey, the types of assessments available were limited to those on which the patient could report. Diagnosis and subtype of MS were not confirmed with patient records, and commonly reported clinical measures such as the EDSS could not be included. The present study focused on HRQoL and self-reported impairment and did not analyze healthcare resource use or costs; the relationship between symptom clusters and these important health outcomes may be different than their relationship with HRQoL or impairment to work or activities.

## 5. Conclusions

Symptoms in RRMS are strongly related to patient-reported outcomes of HRQoL and work productivity. The symptoms seem to cooccur in clusters, and future studies should attempt to confirm whether symptoms cluster in similar ways among other samples of patients or if the presence of certain symptoms or clusters identifies a particular subtype within RRMS. It appears that only some clusters account for variation in patient outcomes; pain, stiffness, and muscle spasms considered as a group seem to interfere with the ability of the individual to do the physical things they want and need to do; depression is predictably associated with poorer mental health, and a greater number of symptoms impact both mental health and one's overall assessment of how good one's health is. Assessments of MS should incorporate patient experience of symptoms and impact on HRQoL in addition to clinical measures of disease progression and disability such as EDSS for a fully comprehensive evaluation of treatment interventions.

## Figures and Tables

**Table 1:** Characteristics of the sample.

Demographics	*n* (%)
Age, mean (SD)	49.3 (11.5)
Female	314 (70.2%)
Race	
White	345 (77.2%)
Black	64 (14.3%)
Hispanic	28 (6.3%)
Asian	5 (1.1%)
Other	5 (1.1%)
Employed	182 (40.7%)
College graduate	164 (36.7%)
Have health insurance	407 (91.1%)

General health characteristics	*n* (%)

BMI	
Underweight	9 (2.0%)
Normal weight	132 (29.5%)
Overweight	138 (30.9%)
Obese	162 (36.2%)
Decline to answer	6 (1.3%)
Exercise in previous month	247 (55.3%)
Smoke cigarettes	103 (23%)
Drink alcohol	260 (58.2%)
Charlson comorbidity index, mean (SD)	0.40 (0.98)

MS characteristics	*n* (%)

Years diagnosed with MS, mean (SD)	12.3 (9.0)
Treatment status	
Currently treated	360 (80.5%)
Disease modifying treatment only	299 (66.9%)
Disease modifying treatment w/dalfampridine/other	25 (5.6%)
Only dalfampridine/other	36 (8.1%)
Formerly treated	48 (10.7%)
Never treated	39 (8.7%)
Self-reported severity of MS	
Mild	194 (43.4%)
Moderate	231 (51.7%)
Severe	22 (4.9%)

Note: disease-modifying medications include interferon 1a or 1b, glatiramer acetate, fingolimod, mitoxantrone, or natalizumab. Dalfampridine is a treatment to improve walking difficulties in MS and is not disease modifying.

**Table 2:** Symptoms among survey respondents with relapsing-remitting multiple sclerosis by perceived severity.

Symptom	Total (*N* = 447)	Mild (*N* = 194)	Moderate or severe (*N* = 253)
Fatigue	357 (79.9%)	147 (75.8%)	210 (83%)
Difficulty balancing/walking	324 (72.5%)	106 (54.6%)	218 (86.2%)∗∗∗
Numbness	284 (63.5%)	107 (55.2%)	177 (70%)∗∗
Difficulty remembering	230 (51.5%)	77 (39.7%)	153 (60.5%)∗∗∗
Pain	230 (51.5%)	62 (32%)	168 (66.4%)∗∗∗
Muscle spasms	223 (49.9%)	60 (30.9%)	163 (64.4%)∗∗∗
Difficulty concentrating	195 (43.6%)	59 (30.4%)	136 (53.8%)∗∗∗
Vision problems	192 (43%)	75 (38.7%)	117 (46.2%)
Urinary incontinence/urgency	183 (40.9%)	52 (26.8%)	131 (51.8%)∗∗∗
Depression	175 (39.1%)	62 (32%)	113 (44.7%)∗∗
Stiffness	164 (36.7%)	42 (21.6%)	122 (48.2%)∗∗∗
Dizziness	151 (33.8%)	47 (24.2%)	104 (41.1%)∗∗∗
Constipation	135 (30.2%)	40 (20.6%)	95 (37.5%)∗∗∗
Mood swings	131 (29.3%)	52 (26.8%)	79 (31.2%)
Irritability	122 (27.3%)	39 (20.1%)	83 (32.8%)∗∗
Sexual dysfunction	120 (26.8%)	36 (18.6%)	84 (33.2%)∗∗∗
Difficulty with speech	112 (25.1%)	37 (19.1%)	75 (29.6%)∗
Tremor	68 (15.2%)	17 (8.8%)	51 (20.2%)∗∗∗
Itching	67 (15%)	19 (9.8%)	48 (19%)∗∗
Swallowing problems	65 (14.5%)	16 (8.2%)	49 (19.4%)∗∗∗
Hearing loss	42 (9.4%)	12 (6.2%)	30 (11.9%)∗
Breathing problems	34 (7.6%)	5 (2.6%)	29 (11.5%)∗∗∗
Diarrhea	32 (7.2%)	11 (5.7%)	21 (8.3%)
Seizures	7 (1.6%)	2 (1%)	5 (2%)

Note: ∗*P* < 0.05; ∗∗*P* < 0.01; ∗∗∗*P* < 0.001.
